# An Itch on the Move: A Diagnostic Dilemma of Cutaneous Serpiginous Eruption

**DOI:** 10.7759/cureus.102751

**Published:** 2026-01-31

**Authors:** Aditi Dabhra, Arushi Nanda (Gakhar), Sanjeev Gupta, Aneet Mahendra, Nikita Gupta

**Affiliations:** 1 Dermatology, Maharishi Markandeshwar (MM) Institute of Medical Sciences and Research (Deemed to be University), Ambala, IND

**Keywords:** creeping eruption, cutaneous larva migrans, hookworm, parasitic disease, pruritic serpiginous lesions

## Abstract

Cutaneous larva migrans is a common parasitic dermatosis, typically affecting the feet and lower extremities, but involvement of atypical anatomical sites may lead to diagnostic delay. We report a 40-year-old woman who presented with an intensely pruritic, migratory serpiginous eruption that sequentially involved the ear, neck, shoulder, and scapular region over one month. A detailed exposure history, characteristic migratory morphology, and supportive dermoscopic findings established the diagnosis of cutaneous larva migrans. The patient responded well to systemic albendazole with adjunctive topical ivermectin. This case highlights that cutaneous larva migrans should be kept in the differential of a creeping migratory lesion irrespective of site.

## Introduction

Cutaneous larva migrans is a parasitic dermatosis resulting from penetration of hookworm larvae into human skin following exposure to soil contaminated with animal feces [1]. The term cutaneous larva migrans was first introduced by Crocker in 1893 [2]. The condition is predominantly encountered in tropical and subtropical regions and is also frequently observed in individuals who have traveled to these endemic areas [3]. This condition is described in the literature by several alternative terms, including sandworm disease, creeping eruption, plumber’s itch, and epidermatitis linearis migrans [4].

The life cycle involves eggs shed in animal feces that hatch in soil, releasing rhabditiform larvae, which mature into infective filariform larvae capable of penetrating skin. Humans are accidental or "dead-end" hosts, as the larvae lack the enzymes required to breach the basement membrane and enter the dermis or circulation, resulting in confinement to the epidermis without further maturation. Lesion migration occurs due to active larval movement within the superficial epidermis mediated by proteolytic enzymes, producing the characteristic serpiginous track.

Diagnosis is primarily clinical, supported by a typical exposure history and morphology, while first-line treatment includes oral ivermectin or albendazole, which rapidly halt larval migration and relieve symptoms. The lesions are intensely pruritic and can present as single or multiple tracks and may appear linear, serpiginous, or intertwined. The extent of these tracks is highly variable, at times extending to several centimeters, with a width of approximately 2-4 mm [5]. Although clinical features are usually characteristic, atypical morphology and sites may delay diagnosis, cause psychological stress, and increase the patient's financial burden.

We describe a 40-year-old woman with an intensely pruritic, erythematous lesion that appeared sequentially over the ear, neck, shoulder, and scapular region over one month, mimicking inflammatory and parasitic dermatoses. The elusive nature of its progression initially obscured diagnosis. Recognition of its characteristic evolution ultimately led to the identification of cutaneous larva migrans, with prompt therapeutic response.

## Case presentation

**Figure 1:**
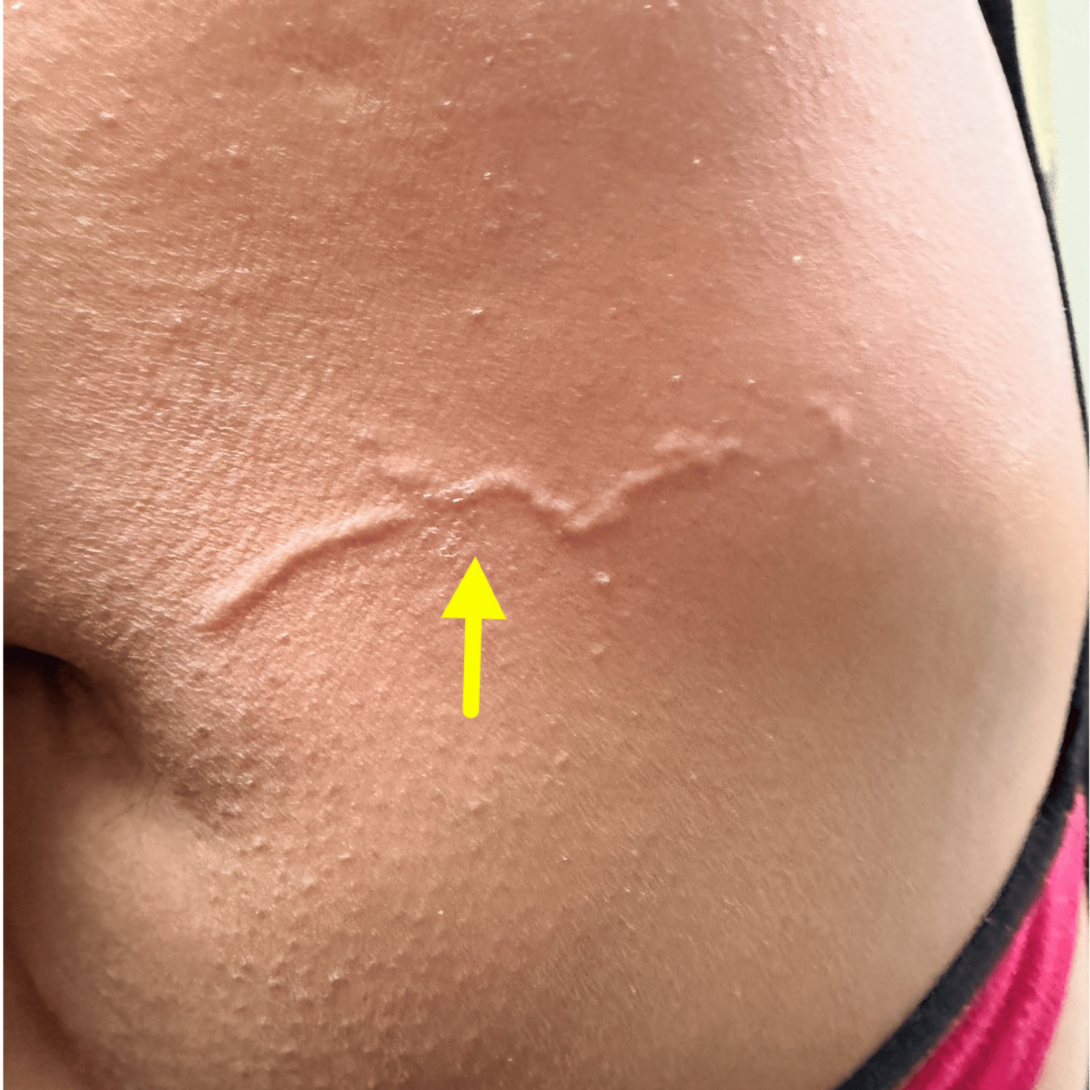
Clinical photograph showing a well-defined, linear to serpiginous, slightly elevated track over the lateral aspect of the back (yellow arrow), corresponding to the migratory lesion described clinically.

A 40-year-old female presented with a one-month history of an intensely pruritic cutaneous eruption that was progressively migrating. The lesion first appeared as an itchy erythematous plaque over the ear one month earlier, subsequently resolving partially before reappearing over the neck, then the shoulder, and finally localizing over the scapular region over one month. Over time, the lesion evolved from an erythematous plaque into a serpiginous, migrating track, reaching approximately 7-9 cm in length over the back (Figure 1).

The eruptions were intensely pruritic, accompanied by redness and a peculiar moving sensation of the lesion. The patient went to a local practitioner, who initially diagnosed the lesion as urticarial dermatitis and prescribed topical corticosteroids and antihistamines, but the patient was not completely relieved. There was an absence of systemic symptoms, lymphadenopathy, and mucosal involvement. There was no history of similar lesions, atopy, or contact with affected individuals. On detailed exposure assessment, the patient reported bathing in a local pond three days prior to symptom onset. The patient removed her slippers before entering the pond. There was no history of walking barefoot on damp soil or sand, lying on beaches, occupational soil exposure, contact with stray animals, or recent travel to endemic coastal areas.

**Figure 2:**
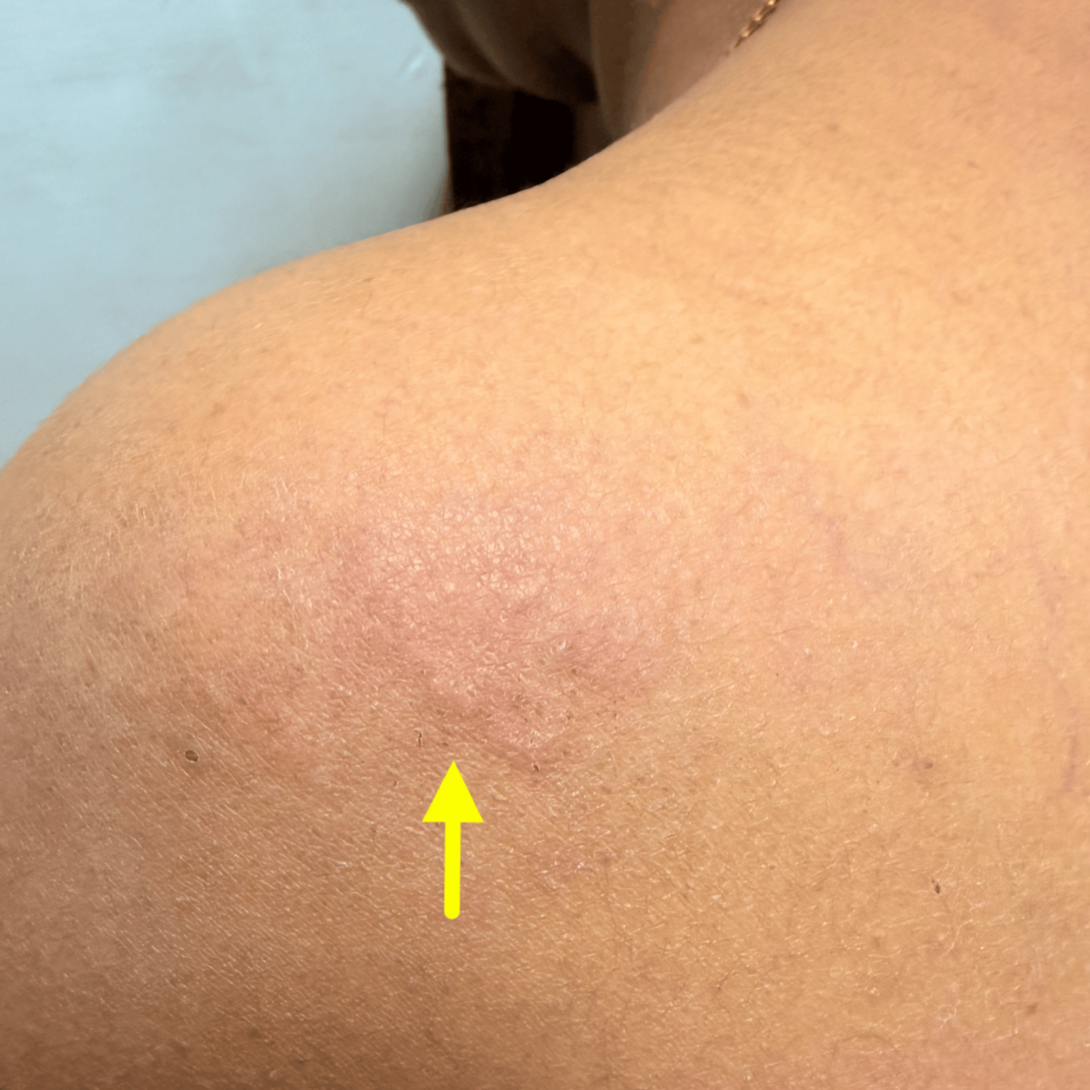
A single, ill-defined, oval to irregular post-inflammatory hyperpigmentation is noted over the shoulder region of size approximately 3–4 cm in diameter (yellow arrow).

Cutaneous examination revealed two active serpiginous lesions at different anatomical sites: a longer, partially resolving serpiginous track measuring approximately 7-9 cm over the lateral aspect of the back (Figure 1), and a shorter, well-defined erythematous, raised serpiginous track measuring 3-4 cm over the scapular region, with surrounding excoriations. Multiple areas of post-inflammatory hyperpigmentation were noted over the neck and shoulder, corresponding to previously involved sites (Figure 2).

**Figure 3:**
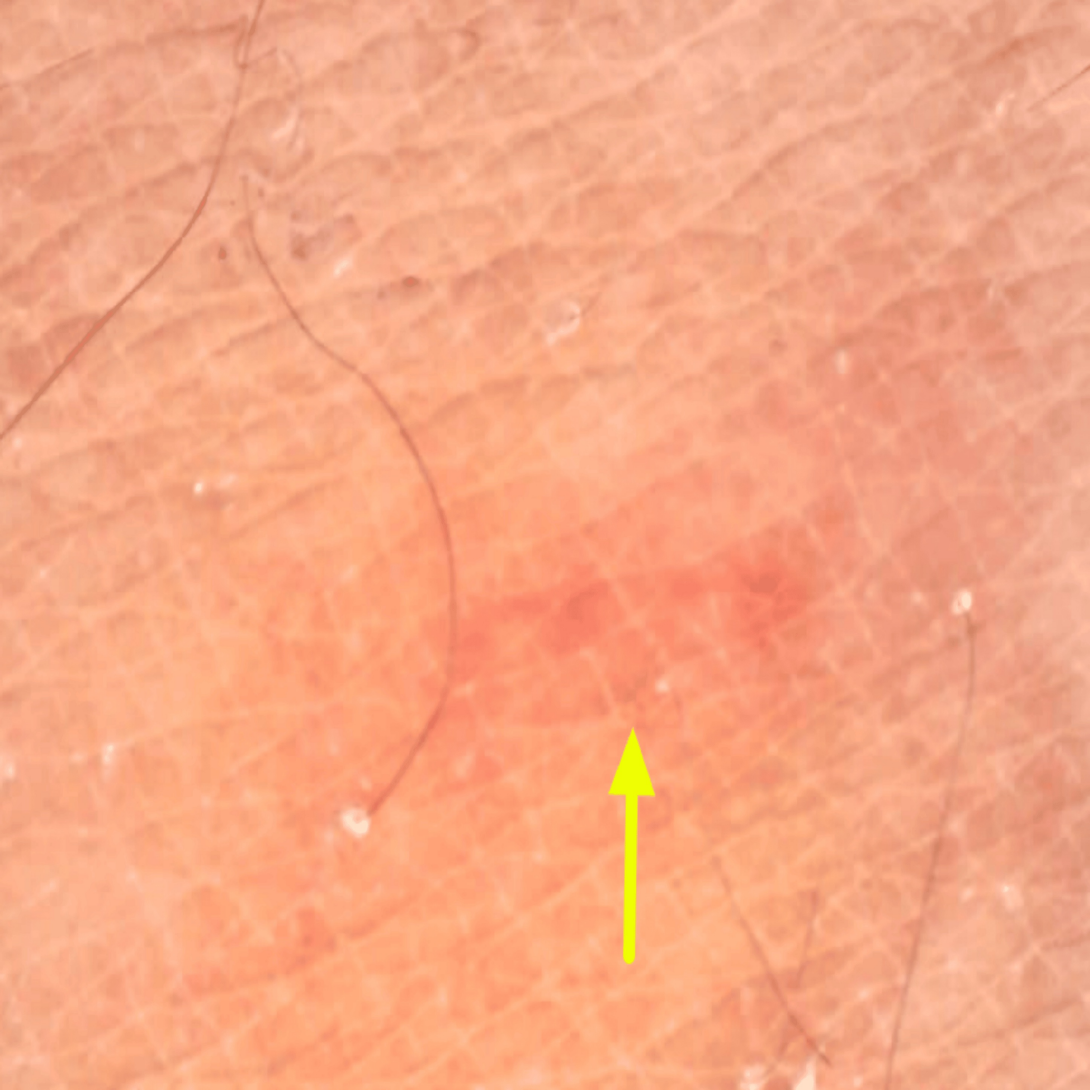
Dermoscopy (40x magnification) revealed a structureless, ill-defined pale pink to erythematous serpiginous track (yellow arrow). No pigment network, globules, serpiginous tracks, or specific vascular patterns are seen. Hair follicles are preserved.

Dermoscopy demonstrated a serpiginous erythematous track corresponding to the active migratory pathway of the larva within the epidermis (Figure 3), supporting the clinical diagnosis of cutaneous larva migrans and aiding exclusion of close differentials such as tinea corporis and urticaria. Correlation of the characteristic migratory morphology, dermoscopic findings, and relevant exposure history led to the diagnosis of cutaneous larva migrans. A skin biopsy was performed to rule out differentials such as larva currens, scabies, tinea corporis, and urticaria, but was inconclusive. The patient was treated with tablet albendazole and topical ivermectin, following which there was rapid resolution of pruritus and gradual clearance of the cutaneous tracks. On follow-up, the lesions had completely resolved without residual scarring or recurrence. The patient remained asymptomatic at subsequent visits. There was no family history of the disease, and no similar signs or symptoms were reported among neighbors. 

## Discussion

In cutaneous larva migrans, infective filariform larvae of animal hookworms penetrate intact or macerated human skin through hair follicles, sweat ducts, or minor abrasions after contact with contaminated soil or sand. As humans are accidental hosts, the larvae do not multiply within the skin; instead, they migrate within the superficial epidermis by secreting proteolytic enzymes such as hyaluronidase, advancing a few millimeters to centimeters per day and provoking a localized inflammatory and eosinophil-rich hypersensitivity response. Cutaneous larva migrans is typically confined to the feet or lower extremities; however, involvement of atypical sites such as the buttocks, genital region, chest, flanks, umbilical area, and interdigital spaces has also been documented [1,5]. Involvement of the ear and upper back, as observed in this case, is distinctly uncommon and may obscure timely diagnosis.

There are also reports of different types of unusual presentations such as folliculitis, bullous eruptions, and even eczematous lesions. Among atypical manifestations of cutaneous larva migrans, hookworm folliculitis represents the most frequently documented. Hypersensitivity reactions may occur, and in some cases, pulmonary eosinophilia consistent with Löffler’s syndrome has been reported [6].

The closest differential is larva currens, which demonstrates a rapid migration course occurring at a rate of several centimeters per hour as compared to several centimeters per day as seen in cutaneous larva migrans. In the present case, the patient exhibited a slowly progressive pattern, with lesion migration occurring over several days rather than hours. Dermoscopy, as previously described by Somasundaram et al. [7], revealed brownish linear serpiginous tracts suggesting a larval body. In our case, dermoscopy revealed a serpiginous, erythematous track, which may represent an inflammatory and hypersensitivity response of the host to migrating larval antigens within the superficial epidermis.

Systemic therapy with albendazole, thiabendazole, or ivermectin constitutes the mainstay of treatment for cutaneous larva migrans. Adjunctive topical therapies, including metronidazole, ivermectin, or thiabendazole formulations, may also be employed [8]. Additionally, topical application of 5% permethrin twice daily for a duration of two weeks has been reported to be effective [9]. The gradual, serpiginous progression and intense pruritus remain the most reliable diagnostic clues. This case is distinguished by involvement of exceptionally uncommon anatomical sites, namely the ear, shoulder, and upper back, which are rarely reported and may obscure timely diagnosis. Dermoscopic evaluation further supported the diagnosis. This case expands the clinical spectrum of cutaneous larva migrans and underscores the importance of maintaining diagnostic suspicion even in atypical locations.

## Conclusions

Cutaneous larva migrans is a commonly encountered parasitic dermatosis; however, involvement of atypical anatomical sites can obscure its diagnosis and lead to unnecessary delay in appropriate management. This case highlights an unusual presentation involving the ear, shoulder, and upper back, emphasizing the importance of careful clinical evaluation, detailed exposure history, and recognition of characteristic lesion morphology.

Early identification and prompt antiparasitic therapy result in rapid symptom resolution and excellent outcomes. Clinicians should maintain a high index of suspicion for cutaneous larva migrans in patients presenting with intensely pruritic, serpiginous cutaneous eruptions at uncommon sites, as timely diagnosis not only alleviates patient distress but also prevents prolonged and unwarranted treatments.
